# Improving Performance in Complex Surroundings: A Mixed Methods Evaluation of Two Hospital Strategies in the Netherlands

**DOI:** 10.34172/ijhpm.2023.7243

**Published:** 2023-05-06

**Authors:** Erik Wackers, Simone van Dulmen, Bart Berden, Jan Kremer, Niek Stadhouders, Patrick Jeurissen

**Affiliations:** Radboud University Medical Center, Radboud Institute for Health Sciences, IQ healthcare, Nijmegen, The Netherlands

**Keywords:** Hospital Strategy, Quality Improvement, Cost Reduction, Implementation, The Netherlands

## Abstract

**Background:** Hospital strategies aimed at increasing quality of care and simultaneously reducing costs show potential to improve healthcare, but knowledge on real-world effectiveness is limited. In 2014, two Dutch hospitals introduced such quality-driven strategies. Our aim was to evaluate contexts, mechanisms, and outcomes of both strategies using multiple perspectives.

**Methods:** We conducted a mixed methods evaluation. Four streams of data were collected and analysed: (1) semi-structured interviewing of 62 stakeholders, such as medical doctors, nurses, managers, general practitioners (GPs), and consultants; (2) financial statements of both organisations and other hospitals in the Netherlands (counterfactual); (3) national database of quality indicators, and patient-reported experiences; and (4) existing material on strategy development and effects.

**Results:** Both strategies resulted in a relative decrease in volume of care within the hospital, while quality of care has not been affected negatively. One hospital failed to cut operating costs sufficiently, resulting in declining profit margins. We identified six main mechanisms that impacted these outcomes: (1) Quality-improvement projects spur change and commitment; (2) increased coordination between hospital and primary care leads to substitution of care; (3) insufficient use of data and support hinder quality improvement; (4) scaling down hospital facilities is required to convert volume reductions to cost savings; (5) shared savings through global budgets lead to shared efforts between payer and hospital; and (6) financial security for physicians facilitates shift towards quality-driven care.

**Conclusion:** This integrated analysis of mixed data sources demonstrated that the institution-wide nature of the strategies has induced a shift from a focus on production towards quality of care. Longer-term (financial) sustainability of hospital strategies aimed at decelerating production growth requires significant efforts in reducing fixed costs. This strategy poses financial risks for the hospital if operating costs are insufficiently reduced or if payer alignment is compromised.

## Background

**Key Messages**

**Implications for policy makers**

- Hospitals may potentially contribute to containing healthcare costs by seeking better quality of care.
- Scaling down hospital cost structures lag reductions in patient volumes and this is a major risk for the accomplishment of long-term savings.
- Data infrastructure and data sharing between providers may facilitate strategies towards appropriate care delivery.
- Hospitals can generate change by defining mutual goals with key stakeholders and continuously adapting to dynamic local contexts.
- Reimbursement of hospital care may incorporate incentives for appropriate care to guarantee financial security while production growth decelerates.

**Implications for the public**

Appropriate care is often proposed as a solution to gain more effect from the increasingly scarce resources in healthcare. Hospitals may contribute to appropriate care. Two hospitals in the Netherlands have attempted to improve their performance with a quality-driven transformation. Their strategies were characterised by: (1) physician-initiated improvement projects, (2) hospital-payer alignment, and (3) increased coordination with general practitioners (GPs) in their region. Our mixed methods evaluation demonstrates that targeting appropriate care through such an institution-wide strategy may yield short term results in volume reductions. However, longer-term (financial) sustainability is a cause for concern, as internal costs need to be reduced accordingly to maintain profit margins. Challenges lie in scaling down hospital facilities to reduce the fixed cost base.

The dominant position of hospitals in healthcare systems is challenged as care can increasingly be provided outside these institutions.^1^ Hospitals are complex organizations that house a variety of models of care within a single organisation.^2^ As financial pressure increases, policy-makers seek fundamental changes in hospital service delivery, aiming for appropriate care since inefficient services may lead to low value care, reducing both quality and financial sustainability.^3^

 Hospital-initiated strategies that aim to improve quality and reduce costs are attractive and thus sought-after. Examples of such strategies are total quality management, lean, six sigma, and value-based healthcare. Previous case-based research demonstrated that improving quality of care may indeed reduce costs.^4-10^ Although a range of case studies was conducted, evidence on real-world effectiveness across the entire hospital is relatively limited. Contextual factors, such as organisational culture, may account for mixed results across varied settings.^5,6,10^ These studies may be subject to bias, since they are relatively scarce, and predominantly report positive effects, and are often conducted by authors affiliated with the institution.^10^

 Moreover, insights in possible interactions between separate contextual factors are underdeveloped.^11^ Traditional case study evaluations are mostly linear (eg, time series analysis) and have limited generalisability across varying context and settings. Quality improvements are often “facilitated evolutions.”^12^ Earlier work shows a lack of integrated improvement research that combine evaluations of costs, quality of care and implementation.^5^ This study aims to add to current knowledge through the use of a multi-perspective analysis on complex hospital strategies, defined by local dynamic context and a nonlinear evolution. Such approaches to evaluations of complex adaptive systems have proven their value in the context of healthcare.^13-15^

 Two hospitals in the Netherlands have committed to such strategies and have taken explicit steps towards financial sustainability through quality improvement. Fundamental elements of their strategies were (1) hospital and payer alignment, (2) engagement of physicians, and (3) an orientation toward partners in their catchment area.^16,17^ Early self-reported results indicated substantial improvements.^16,18^ However, it is unknown how quality improvement processes were actually undertaken, which factors contributed to their outcomes, and how the effects translate into quality and costs at the hospital level.^19^ Approximately five years after the initiation of their reforms, we examined the extent to which both hospitals reached these goals. Our primary research question was “How can institution-wide improvement programmes contribute to the aim of reducing costs through quality improvement?” Multiple sources of data, both quantitative and qualitative, were used to examine contexts, mechanisms, and outcomes, following the Medical Research Council’s framework for complex interventions.^20,21^

###  Institutional Context

 Bernhoven is a mid-sized (approximately 380 beds) general hospital situated in the southeast of the Netherlands. The Beatrix hospital, part of Rivas care group, is a small (approximately 220 beds) general hospital in the southwest of the Netherlands. Both hospitals developed their vision for organisational change inspired by a consultancy report “Quality as remedy.”^17^ Both hospitals faced an urgent need for change. Increasing competition between providers and nearby larger (tertiary) centres threatened their future as smaller-sized general hospitals. In the case of Bernhoven, the funding of a new hospital building added to fiscal pressures. In the case of the Beatrix hospital, a planned merger with a large nearby hospital was not approved by the competition authorities.

## Methods

###  Study Design

 We conducted a convergent parallel mixed-methods study to evaluate contexts, mechanisms and outcomes of both hospital strategies. This enabled collection of multiple data sources, both quantitative and qualitative, with iterative cycles of validation and confirmation.^22^ This design complies with the Medical Research Council’s framework for the evaluation of complex interventions.^20,21^ The evaluation was conducted between April 2019 and May 2020.

###  Data Collection

 Four streams of data were collected. The first stream was collected with the aim of assessing implementation mechanisms. Specific quantitative outcomes were collected about quality of care (second stream), financial data (third stream), and additional analyses on care volumes (fourth stream). Two interactive sessions with representatives of both hospitals, insurers, the patients federation, and the Ministry of Health were used to report progress of the evaluation. These sessions were used to validate our findings and identify omissions in the evaluation.

 First, we collected qualitative data through semi-structured interviews. The topic guide was constructed according the Consolidated Framework for Implementation Research.^23^ This framework is suitable for evaluating the implementation process, inner and outer contexts, relevant stakeholders, and outcomes (Supplementary file 1). This framework was used as an extension to the Medical Research Council’s framework, as it consists of a more pragmatic structure for evaluations of complex interventions.^24^ Second, we collected routinely measured hospital quality indicators, which are publicly available through the Health and Youth Care Inspectorate and the National Health Care Institute in the Netherlands.^25,26^ These quality indicators are typically measured per specialty or disease group and are classified according to the Donabedian framework, which uses structure, process and outcome indicators to assess quality.^27^ The number of indicators varies over time and ranges between a maximum of 1932 in 2012 and a minimum of 722 (2018), with the majority being structural indicators on quality of care (Table 1). The National Health Care Institute collects quality indicators and these indicator sets are adjusted and updated annually, resulting in variation across years. In addition, the National Healthcare Map (in Dutch: Zorgkaart Nederland) collects patient experiences from 2008 to 2018 with patients grading hospitals or caregivers on a voluntary basis.^28^ The third data stream was drawn from annual reports and financial statements for both hospitals (2007−2020), which are publicly accessible in the Netherlands.^29^ The main financial indicators that we used for our analysis were hospital revenue, expenses, solvency ratios, and gross and net margins. Trendlines for both intervention hospitals were compared to the (sector) trend of all hospitals in the Netherlands. The final stream of data collection consisted of several additional existing data sources, which were both published and unpublished data of trends in (specific) volumes and quality-improvement projects in both hospitals. Volume trends were analysed by the Netherlands Bureau for Economic Policy Analysis, using quantitative data.^30^ Additional (unpublished) data on individual improvement projects were used as complementary material.

**Table 1:** Overview of Structure, Process, and Outcome Indicators (n) Per Year

**Year**	**2010**	**2011**	**2012**	**2013**	**2014**	**2015**	**2016**	**2017**	**2018**
Structure	757	1674	1826	1815	782	672	823	738	579
Process	31	52	72	71	94	94	114	98	93
Outcome	14	17	34	34	27	27	56	52	50
**Total**	**802**	**1743**	**1932**	**1920**	**903**	**793**	**993**	**888**	**722**

###  Recruitment Process and Sampling Strategy

 The board of directors of both hospitals set up contacts with the strategy programme managers. Key participants were identified by the programme managers (which were interviewed as well) of both hospitals and through purposive sampling in interviews. Our aim was to conduct interviews across the full organization, targeting a variety of medical departments and support staff. External stakeholders (ie, general practitioner [GPs], healthcare insurers, and consultants) were contacted to include multiple perspectives on the transformation programmes. Potential participants were approached by email and a reminder was sent after two weeks. All participants provided written informed consent for this study. We interviewed 62 key stakeholders: medical doctors (n = 19), nurses (n = 5), hospital managers (n = 15), GPs (n = 7), primary care managers (n = 3), external consultants (n = 4), patients (n = 9), and insurer managers (n = 5). Of these stakeholders, five held dual functions (eg, medical doctors and managers). Interviews were held between May 2019 and April 2020. Interviews were conducted concurrently and iteratively to inform subsequent interviews. Supplementary file 2 provides an overview of all the interviewed stakeholders.

###  Data Analysis

 Qualitative interviews were transcribed ad verbatim and analysed thematically in ATLAS.ti (version 8.4.20). The first ten interviews were analysed independently by three researchers to achieve consensus on the coding list, enhancing inter-researcher reliability. After agreement was reached, a single researcher analysed the remaining transcripts using the item list. Data were coded deductively using themes that describe features influencing complex implementation processes, identified in literature: organizational culture, networks and communication, leadership, resources (financial resources, staffing and workload, time, education and training), evaluation, monitoring, and feedback, and champion.^11^ These themes were used for the identification of contextual factors. Interrelationships between different themes demonstrate overarching mechanisms that “can act as enablers in one implementation setting but barriers in others,” for example the effect of leadership on culture, communication and resources.^11^ The identified mechanisms in this study follow evident interrelationships between themes. Finally, interviewing of respondents also revealed (perceived) qualitative outcomes, which were used as supplementary data to the quantitative findings (eg, patient satisfaction, perceived quality).

 To evaluate the actual effects on quality of care, composite indicators were constructed following the standard-normal transformation methodology.^25,31,32^ Each quality indicator was transformed into a z-score expressing deviation from the population mean:


$$z-score_{i,j}=\frac{X_{i,j}-{\bar{X}}_{j}}{\sigma_{i,j}}$$

where *x*_i,j_ is the value of indicator j for hospital I, and σ is the standard deviation. The composite indicator is an unweighted sum of z-scores, reflecting the average deviation from the mean over indicator set J (ie, no differentiation is made in relative importance of individual indicators). A composite indicator was constructed for all routinely measured structural quality indicators (maximum of n = 1826 in 2012, minimum of n = 579 in 2018), process indicators (minimum of n = 31 in 2010, maximum of n = 114 in 2016), and outcome indicators (minimum of n = 14 in 2010, maximum of n = 56 in 2016) (See Table 1). As the content and number of indicators differ over time, constructing a benchmark z-score allows comparisons of the average relative quality performance over time. Data from all Dutch hospitals were used to calculate z-scores. The mean composite indicator scores of other hospitals in the Netherlands are calculated as well to compare trendlines between the intervention hospitals, and the hospital sector. The score therefore represents the relative performance of a hospital versus all other hospitals. An analytic narrative was constructed in the third data stream using data from annual financial reports, interview data, and external data from desk research. Both hospitals were offset against all Dutch hospitals. As the implementation programmes started in 2014 and 2015, 2012 was chosen as a base year to offset any anticipation effects. The median was used to correct for skewed data.

 Difference-in-differences (DID) models were estimated to compare means and trends in the pre- and post-intervention period.^33^ First, we tested whether mean total expenditures differed after intervention year 2015 in the intervention hospitals compared to control hospitals, after correcting for pre-intervention differences.^34-37^ Next, we tested whether growth rates in total expenditures differed after 2015, correcting for pre-intervention differences in levels and rates of growth between intervention and control hospitals. We repeated the analysis for the part of total expenditures paid by insurers, solvency ratios, profit margins and quality indicators. Quality indicators were analysed separately for structure, process, and outcome quality. Only general hospitals were included in the DID models.

## Results

 The strategies of both hospitals were shaped into a complex multiyear implementation programme, consisting of different elements that cover the entire organisation and its stakeholder alignments. These are summarised in Table 2.

**Table 2:** Elements of Hospital-Wide Improvement Strategies for Bernhoven and the Beatrix Hospital: Similarities and Differences

	**Bernhoven**	**Beatrix Hospital**
Improvement projects	>50 bottom-up improvement projects	>50 bottom-up improvement projects
Organisational model	Reorganisation into four business models^2,16^ Acute care Solution shop Intervention unit Chronic care	No organisational restructuring
Increased coordination of primary care providers	Cooperation with primary care physician organisation	Cooperation with primary care physician organisation
Hospital−payer alignment	Multiyear contracts (global budget) to create financial stability	Multiyear contracts (global budget) to create financial stability
Hospital−physician alignment	Salaried payments for physicians (opposed to fee-for-service)	Revenue redistribution model for physicians that supports volume reduction (90% capitation)
Culture change	Cultural programme to induce a culture focused on quality improvement	Implicit shift towards a culture focused on quality improvement

 Six main configurations demonstrate how the strategy and implementation impacted quality and costs in both hospitals. Qualitative data identified and prioritized these six mechanisms. Primarily, both hospitals aimed to improve quality through improvement projects and through substitution of care to primary care settings (mechanisms 1, 2, and 3). Second, multiyear contracts, scaling down of internal costs, and altered remuneration of physicians were required to guarantee financial security for physicians and the hospital while volume of care declined as a result of the improvement projects (mechanisms 4, 5, and 6). Both hospital strategies were evolving processes: their main goals — improving quality and reducing costs — were not explicitly connected to clear targets that could allow for incremental changes to the initial strategy.

 “*The change process was complex: rather than implementing a clear intervention with clear goals, we tried to establish a mutual sense of direction. The direction was improved care for patients, financially sustainable for society and we respect each other’s roles” *(i29, external consultant).

###  Quality-Improvement Projects Spur Change and Commitment

####  Context

 At the core of both implementation programmes, over 50 projects for quality improvement were implemented. These were bottom-up initiated by physicians. Hospital management facilitated project support, including hiring external consultants. Project proposals required an analysis of impact on quality and costs savings. These projects were then evaluated in pilots. A wide range of improvement projects has been implemented. Some involved shared decision-making and decision aids. Others aimed to coordinate and substitute care, such as optometry tests in primary care, or to decrease length of stay in the hospital for specific patient groups.

####  Mechanism

 The bottom-up approach of quality-improvement projects increased the engagement and commitment of physicians that developed their own care projects. Conversely, nurses experienced a lack of engagement. They perceived a stronger focus by the board on stimulating physicians to develop improvement projects. Another barrier to achieving engagement and commitment to quality projects was the administrative burden. All projects had to be presented with a business case demonstrating potential quality gains and savings, which were deemed time-consuming. Nevertheless, this approach was helpful in translating the abstract goal of improving quality to more tangible actions. The envisioned new culture — moving away from production-driven care to quality-driven service — became embedded into the practice of everyday care delivery.

 “*The projects have contributed to cultural change, in my opinion, because everyone was involved and was having a positive experience. This is motivating and has been an important factor in building a new culture*” (i17, hospital manager).

####  Outcome

 A substantial proportion of the care improvement projects in Bernhoven hospital was targeted at shared decision-making. Interviewed patients did not measurably perceive an increase in shared decision-making, but overall patient satisfaction did increase (Supplementary file 3). The Beatrix hospital focused several projects on substituting care towards GPs.

**Figure 1:**
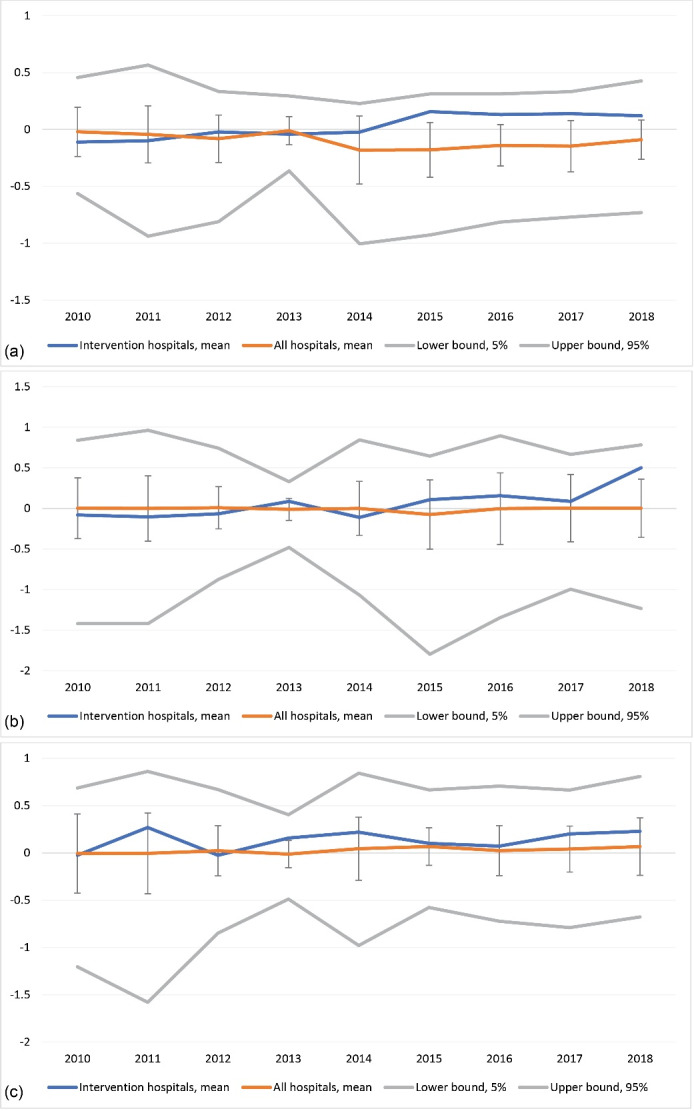
Trendline Z-Composite Score for (a ) Structure, (b) Process, and (c) Outcome Indicators. Error Bars Indicate the Standard Error.

 Indicators on quality of care — structure, process and outcome — show predominantly positive trendlines (Figure 1). These indicators improved slightly over the implementation runtime compared to other hospitals.

 DID models (Tables 3 and 4) show that means for outcome quality were lower in the intervention group in the pre-intervention period, whereas structure and process quality means were similar to other general hospitals. After intervention, process quality significantly improved (Table 3). Similarly, trendlines for process quality had a significantly steeper slope in the intervention group. There were no clear effects on structural and outcome quality.

**Table 3:** Difference-in-Differences Analysis: Comparing Pre-post Intervention Means Between Intervention Group and Non-intervention General Hospitals

	**Insurer-Based Expenditures (Millions)**	**Total Expenditures (Millions)**	**Solvency ** **Ratio**	**Profit ** **Margin**	**Structural Quality**	**Process ** **Quality**	**Outcome Quality**
Intervention group	-105^c^ (9.28)	-92.1^c^ (15.30)	-0.05^c^ (0.01)	-0.003 (0.004)	-0.034 (0.04)	-0.045 (0.04)	0.133^b^ (0.05)
Post intervention period	81.1^c^ (12)	96.8^c^ (19.70)	0.153 (0.08)	-0.042 (0.03)	0.037^a^ (0.02)	-0.017 (0.02)	0.012 (0.02)
Intervention * post intervention period	-32.5 (19.50)	-47.2 (25.60)	-0.102 (0.09)	0.04 (0.03)	0.125 (0.22)	0.205^b^ (0.07)	0.129 (0.17)
Constant	213^c^ (5.61)	284^c^ (9.42)	0.171^c^ (0.003)	0.015^c^ (0.002)	0.01 (0.01)	0.015 (0.01)	-0.016 (0.01)
N	1052	1064	1061	1062	459	595	595
F	81.15	30.11	5.98	1.52	1.82	8.38	3.58
R^2^	0.06	0.03	0.01	0.004	0.01	0.01	0.01

Standard error reported in round brackets. ^a^ 5%, ^b^ 1%, ^c^ 0.1%.

**Table 4:** Difference-in-Differences Analysis: Comparing Pre-post Intervention Trends Between Intervention Group and Non-intervention General Hospitals

	**Insurer-Based Expenditures (Millions)**	**Total Expenditures (Millions)**	**Solvency ** **Ratio**	**Profit ** **Margin**	**Structural Quality**	**Process Quality**	**Outcome Quality**
Pre-intervention control group trend	9.224^c^ (2.18)	14.3^c^ (3.53)	0.009^b^ (0.003)	0.001 (0.001)	0.006 (0.01)	-0.008 (0.01)	-0.0004 (0.01)
Intervention group level	-105^c^ (8.55)	-92^c^ (14.20)	-0.055^c^ (0.01)	-0.002 (0.004)	-0.033 (0.04)	-0.047 (0.06)	0.127^b^ (0.05)
Intervention group trend pre-intervention	1.222 (1.68)	-0.361 (2.76)	0.006 (0.01)	-0.004 (0.003)	0.001 (0.003)	0.002 (0.002)	0.001 (0.004)
Intervention group trend post-intervention	-1.427 (1.79)	-4.321 (2.46)	-0.001 (0.002)	-0.001 (0.001)	0.012 (0.02)	0.021^c^ (0.01)	0.015 (0.02)
Constant	167^c^ (11.20)	213^c^ (17.80)	0.129^c^ (0.02)	0.008 (0.01)	-0.026 (0.03)	0.071 (0.04)	-0.014 (0.06)
N	1052	1064	1061	1062	459	595	595
F	70.92	19.68	27.30	8.66	39.90	1.40	1.84
R^2^	0.08	0.02	0.04	0.01	0.01	0.003	0.01

Standard error reported in round brackets. ^a^ 5%, ^b^ 1%, ^c^ 0.1%.

###  Increased Coordination Between Hospital and Primary Care Leads to Substitution of Care

####  Context

 Multiple projects involved substitution of hospital care to primary care providers. This may reduce unnecessary hospital visits and lead to cost reduction.^38^ Thus, both hospitals closely collaborated with the overarching cooperatives of primary care physicians (care groups). GPs were closely and actively involved in the implementation phase.

####  Mechanism

 An important prerequisite for reducing unnecessary care in the hospital and delivering care closer to home is increased coordination between hospital and GPs. Both hospitals traditionally have strong relations with their respective primary care organisations. This was considered a facilitator for improved coordination and collaboration. GPs stated that their involvement in the development of the care improvement strategies and the implementation programmes increased engagement and trust. Whereas interaction between specialists and GPs was traditionally experienced as hierarchical, the programmes contributed to the perception of communication at peer-level. Through better collaboration and coordination efficiency in the care chain improved, benefiting patients as well.

 “*Sometimes a brief consultation with the hospital is necessary, but they do not need to repeat the entire patient process …. Our collaboration has improved a lot, to avoid repeating actions. Unnecessary actions put a strain on patients” *(i40, GP).

 However, as a result of a shifting volume towards primary care, GPs experienced an increased workload. In some cases, GPs were compensated, but in others they also indicated a lack of compensation for their efforts. As GPs play a pivotal role in the strategies, some interviewees stated that they should also be included in the multiyear contracts.

####  Outcome

 The number of unique patients that received care at their GP practice increased, 0.4% in the Beatrix hospital region and 3.5% in the Bernhoven region was reported.^39^ Combined with reductions in hospital patients, an increase in unique patients may be an indication of substitution. The idea was that substitution of care should not affect quality of care in a negative way. We have collected some evidence on quality of care for specific projects. Patients with diabetes mellitus type 2 and hypothyroidism who were redirected to their GP did show outcomes that were on par with hospital care, although patient satisfaction declined slightly.^40^

###  Insufficient Use of Data and Support Hinder Quality Improvement

 Data and support were identified as barriers to the improvement process. The exemplar was the lack of data infrastructure for coordination and collaboration between hospitals and primary care. Ideally, GPs and medical specialists work in the same electronic health record, but current systems are not compatible, increasing administrative burdens.

 The importance of project support was emphasised in both hospitals. At Bernhoven, specific resources were allocated to project support, but these were scaled down during the runtime of the implementation programme. In contrast, in the Beatrix hospital, project support was incorporated at the heart of the strategy, enabling new projects towards the end of the implementation programme.

###  Financial Security for Physicians Facilitates Shift Towards Quality-Driven Care

####  Context

 In the Netherlands, specialists are either self-employed within a group practice (39%), salaried (49%) or a combination of both (12%).^41^ Bernhoven and Beatrix hospital mainly rely on self-employed physicians. Both hospitals altered physician payment structures. Hospital management and medical staff in Bernhoven decided for the boldest approach: self-employed doctors became salaried physicians, aiming to eliminate most production incentives.^16,42^ Physicians in the Beatrix hospital remained self-employed, but their income was no longer tied to volume; physicians were guaranteed a base income and were rewarded for engaging in quality-improvement initiatives (for up to 10% of their income). In both hospitals, physicians would not be negatively financially affected when volumes decreased.

####  Mechanism and Outcome

 Financial incentives for physicians were altered in both hospitals. Several respondents indicated that detaching income from production can incentivise for a reduction in low-value care. Some stated that now they were no longer affected in their professional assessment by financial incentives.

 “*It was easier to not admit patients into the hospital when doctors were no longer dependent on production for their income …. In contrast, doctors used to compete each other for a patient’s admission because their department would be remunerated” (*i4, MD and hospital manager).

 Physicians experienced financial security, which accelerated improvement projects aimed at reducing low-value care. Nevertheless, the transition from self-employed to salaried status initially met with high resistance at Bernhoven. Physicians feared loss of power and an increased dependency on hospital management. The self-developed concept of medical leadership, where doctors combined clinical and managerial functions, was thought to prevent such dependence. Physicians at Beatrix hospital indicated that they now were better incentivised to improve quality since the new redistribution model rewarded them for doing so.

###  Scaling Down Hospital Facilities Is Required

####  Context

 Bernhoven incorporated a new organisational model.^2^ Instead of structures emphasising the different medical specialties, four business units were created based on workflow processes: (1) diagnosis and decision making, (2) intervention unit, (3) acute care unit, and (4) a chronic care unit.^16^ The assumption was that organising the hospital according to workflow functions would harmonize processes of care and, ultimately, lead to efficient service delivery. For example, blocks of planned surgery appointments could be scheduled more efficiently, and acute care was strengthened with additional medical expertise to avoid unnecessary downstream costs. Beatrix did not restructure their organisational model, but aimed to scale down internal cost structures.

####  Mechanism

 To reduce costs, the decreasing volumes of care eventually require reducing the cost-structure of the hospital. However, scaling down (semi) fixed-costs takes time and thus the savings lag actual volume reductions. Combining quality initiatives with reorganising workflow processes might allow scaling down capacity. For example, Bernhoven’s reorganisation into four business units was perceived to contribute to a more streamlined care delivery process.

 “*The business units fit the philosophy and culture of the Dream programme: a shared responsibility among departments benefiting the patient with a more holistic view” *(i17, hospital manager).

 Although the Beatrix hospital did not fundamentally reorganise its core processes, substantial efforts were made to reduce fixed costs. An entire nursing ward was closed as a result of fewer hospitalisations. However, significant drops in volume are necessary to be able to close an entire ward and the limited scale of these regional hospitals is a barrier since a minimum necessary scale exists.

 “*A barrier for small hospitals is the amount of fixed costs that cannot be reduced…. Some departments cannot be scaled down in terms of staffing if you wish to have a continuous service available. Surgeons and their subspecialties, need sufficient staffing”* (i12, MD and hospital manager).

####  Outcome

**Figure 2:**
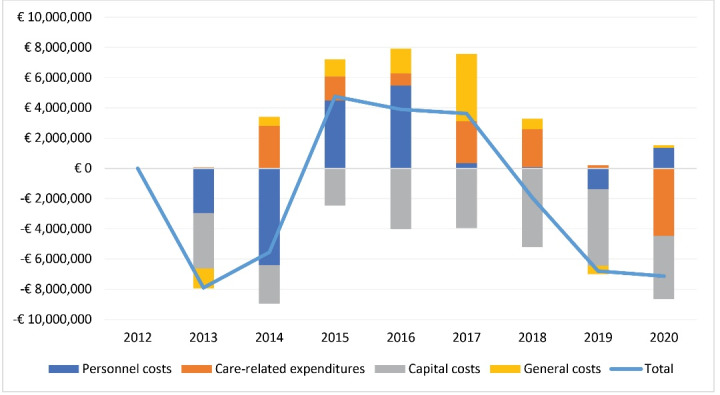
Bernhoven Operating Expenditure Growth Relative to the Median of All Hospitals (2012 Base Year). Total costs are a sum of personnel, capital, general, and care-related costs.

 Annual reports for Bernhoven show reductions in operating costs relative to other hospitals (Figure 2). While capital costs have declined, personnel expenditure demonstrate a relative increase in 2020, compared to other hospitals. Variable care-related costs witness substantial decline from a top in 2017 which correlates with the lower volumes. As Beatrix hospital is part of a larger organisation that also includes long-term-care provision (Rivas), these trendlines cannot be isolated for this hospital.

###  Multiyear Shared Savings Arrangements Under Global Budgets May Increase Alignment Between Payers and Hospitals

####  Context

**Figure 3:**
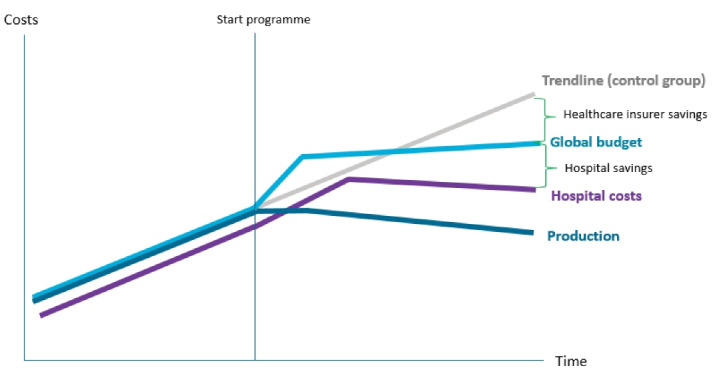
Conceptual Model Multiyear Global Budget.

 In the Netherlands, hospital payment is case-based (diagnosis−treatment combinations).^41^ Price and volumes of these diagnosis−treatment combinations are negotiated between insurance companies and providers.^43^ It is assumed that this may provide doctors with incentives for overutilization, leading to unnecessary care volumes. Bernhoven and Beatrix hospital came up with alternatives. They formed an alliance with the largest healthcare insurers in their areas and complied to a multiyear global budget contract with a runtime of five years.^16^ This contract included accountability clauses to ensure accessibility and quality of care (ie, to prevent undertreatment). The contract was based on a *shared savings *rationale (Figure 3): insurers pay for necessary upfront investments followed by multiyear flat real growth. Over the contract period, payers pay less than trendline cost growth and thus they collect savings. The multiyear agreement provides the hospitals with financial security while transforming their organisation to reduce their structural cost base. Hospitals may benefit financially if they succeed in reducing (fixed) hospital costs and thus collect their part of the shared savings rationale.

####  Mechanism

 The multiyear global budget provided financial security for structural changes in both hospitals. Both strategies required upfront financial investments, which were incorporated into the arrangement. Thinking was that as the strategies aimed to reduce volumes, revenue streams would over time start to decline. Financial security should also engage physicians. The psychological basis of the arrangement between health insurers and hospitals was built on a win-win perspective in which savings were ‘equally’ shared. Cooperative mentality was perceived as a step forward from preceding rivalrous negotiations.

 “*The programme truly gained momentum when two main insurers were committed to the agreement: cost savings would be shared and reallocated to benefit policy holders and reinvested into the hospital …. This created a sense of partnership as opposed to the insurer cream-skimming the hospitals’ efforts”* (i36, healthcare insurer).

 However, the room for volume and cost reductions is not infinite. Besides, uncertainty over upcoming negotiations was present among several stakeholders toward the end of the contract period. This increased tensions between health insurers and the hospital (in the case of Bernhoven). Power asymmetry exists (Ratchet effect): if the insurer decides to return to case-based reimbursements, the hospital might practically not be able to return to former levels of production, threatening the financial viability of the organisation.

####  Outcome

 An important aim was to reduce low-value care. Both Bernhoven and Beatrix hospital managed to decrease their volumes of care delivery compared to a control group of hospitals. The Beatrix hospital decreased its relative volume by 7% in 2017; Bernhoven achieved a whopping 13% decrease.^39^ Additional analyses showed no indication of patients in either region visiting other hospitals (spillover effects).^39^ A key factor is the extent to which such substantial volume reductions result in reductions in operating costs.

 “*We managed to reduce the volume of care, but did we also save costs? The health insurers are saying, ‘The hospital is still too expensive, since the agreed cost reductions were not reached’”* (i35, MD and hospital manager).

**Figure 4:**
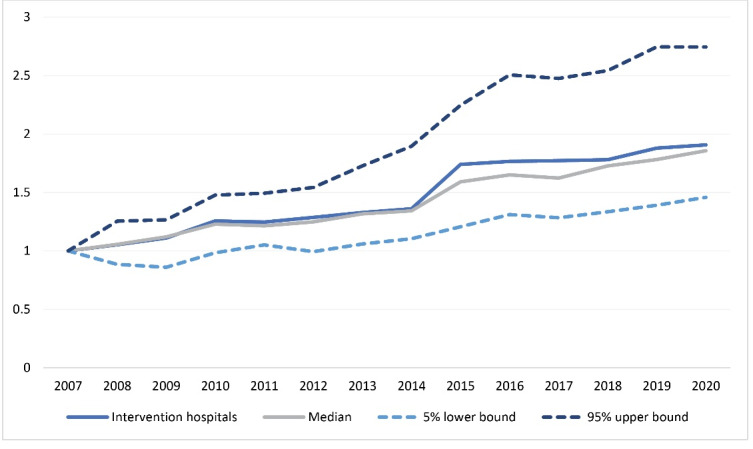
Hospital Revenue (ie, Insurer Expenses) of Both Intervention Hospitals Compared to Counterfactual Based All Other Hospitals in the Netherlands.

 Multiyear global budgets did not lead to reductions in hospital revenue (ie, insurer costs) in the average of both intervention hospitals, compared to other Dutch hospitals (Figure 4). Hospital revenue showed a relative increase in the early years of implementation (upfront investments) after which costs returned to, but not underneath, the level of the other hospitals. This indicates that from the payer perspective, the strategy did not significantly result in lower relative hospital expenses, despite reductions in volume of care. However, the latter might actually lead to a reduction in their own payments from the risk-adjustment funds that partly relate to hospital volume. The profit margins at Bernhoven also were on a negative path, but Rivas (Beatrix hospital) shows an upward trend since 2016 (Supplementary file 4). The latter does includes the whole care group and we cannot separate hospital results from those of the elderly care units.

Tables 3 and 4 show DID models estimating the effects on hospital revenue (insurer-based expenditures), solvency ratio, and profit margins. Insurer-based expenditures, total expenditures and solvency ratios were lower in the intervention group as compared to other general hospitals (pre-intervention). No significant differences pre-intervention indicate that the parallel trends assumption holds.^33^ The intervention group experienced lower mean expenditures in the post intervention period, but not significantly. Similar results ensue from the DID analyses estimating trends: Baseline values were lower in the intervention group, and trends follow a reduced expenditure growth rate, but not significantly (Table 4). Solvency ratios and profit margins demonstrated no clear differences in trends. These results are qualitatively similar to the trend analyses.

## Discussion

 This study hypothesized six pivotal mechanisms that potentially contribute to quality improvement and cost reduction. First, actual improvements in patient care were achieved through active, bottom-up engagement of professionals in multiple quality projects. Physicians developed and initiated quality-improvement projects that contribute to a strong base of support. Second, both hospitals also managed to substitute care to GPs; communication between primary and secondary care was considered positive and improved. Third, insufficient use of data and support hinders quality improvement. This barrier was apparent in the lack of data infrastructure for primary and hospital care coordination as well as resources for data support. Fourth, in both hospitals, reducing financial incentives for physicians to increase the volume of care was deemed important. Stakeholders perceived a greater focus on quality of care, opposed to volumes. Fifth, scaling down hospitals may convert volume reductions to costs savings. Although both hospitals managed to scale down their organisation, additional efforts are needed to keep pace with volume reductions. This seems to be especially the case at Bernhoven, which witnessed declining profit margins. Sixth, multiyear global budget agreements between payers and hospitals may contribute to decelerating volume growth through financial security. However, concerns are raised whether this strategy is sustainable beyond the contract period, as a result of a lag in the reduction of internal costs.

 Culture was shifted towards quality-based and patient-centred care through a combination of elements of the strategy, such as quality-improvement projects, financial incentives, and culture workshops. The institution-wide implementation of both strategies contributed to their success. Hospital performance was improved through engagement of all stakeholders, facilitating the evolution of a multiyear implementation programme, and strong clinical and managerial leadership.

###  Strengths and Limitations

 The main strength of this study is triangulation of data sources. This allowed us to evaluate hospital strategies that transcend traditional siloes, such as primary and secondary care and provider-payer divisions. The integrated use of methods allowed us to draw conclusions beyond simple outcomes such as cost and quality of care.^7^ However, this does not imply that our findings, as described in six mechanisms, can be used as blueprints for all future hospital improvement programmes. These mechanisms are useful but, ultimately success is related to the engagement of stakeholders within the system and strategy fit to the local context.

 An important limitation to our study is that net effects are difficult to discern. As these strategies are complex and involve multiple elements at different levels within and outside the organisation, we cannot assume causality between specific outcomes we observe and specific elements. Furthermore, our data on costs and quality outcomes had some limitations.

 Explanatory power of our DID models was relatively low, due to the limited number of observations (approximately 70 hospital organisations) in the Netherlands. Furthermore, the start of the improvement programme defined the pre- and post-intervention periods, whereas these interventions were multifaceted (eg, not all quality improvement projects were initiated during the first year). Nevertheless, the explanatory analyses were in line with descriptive trends, ie, insignificantly lower expenditure growth, no evident effects on other financial indicators and limited, positive effects on quality between the intervention hospitals and other hospitals were observed. Given the variance within the control group, the Dutch hospital sector may have been inherently underpowered to detect real effects on quality or costs. Moreover, patient satisfaction scores were subject to selection bias. Some elements are difficult to capture in indicators such as patient-centredness. Furthermore, available quality indicators were subject to large variation between years, which limited comparisons over time for individual variables and specific sets of indicators. To correct this, we constructed an average benchmark z-score. This benchmark score may level out positive and negative outliers. In theory, variance in quality indicators may increase as a result of the strategic programs, but this was beyond the scope of this research. Therefore, effects on quality should be interpreted with caution. Although we interviewed patients, it was difficult to ascribe personal experiences to specific mechanisms. Nevertheless, the broad spectrum of data sources and indicators we used to an extent do mitigate such limitations. The interpretation of results should be integrated, rather than based on individual data streams and analyses.

###  Previous Research

 While several studies have evaluated improvement programmes that target single departments or patient groups, evaluations of institution-wide strategy implementations are much more scarce.^7,8,10,44-46^ Nevertheless, earlier research on organization-wide hospital strategies demonstrated that engagement of staff at all levels was found to be critical for successful implementation.^8,47-50^ These strategies often translate into a multitude of projects on the operational level of hospitals, which can further enhance staff engagement.^9,46^

 Furthermore, a recent systematic review found substitution of hospital care to specialist services in primary care settings to have a positive effect on quality of care.^51^ Although only some projects in Bernhoven and the Beatrix hospital involved this type of substitution, results were similar in terms of costs and quality. The qualitative part of our study also adds that increased coordination between hospital and primary care can facilitate efficient care delivery in both settings.^51,52^

 Evidence on appropriate payment models for specialist physicians remains limited.^42^ The effectiveness of physician payment models seems to depend more on adequate quality and performance indicators than the payment model itself (salaried versus self-employed).^42^ Physician payment models were not the main focus of our study, but both hospitals used different payment models resulting in similar experiences: stakeholders perceived an increased focus on quality rather than volume of care. Another case study on institution-wide hospital improvement showed that cost savings are not generated instantly, but take some time to take effect.^7^ Furthermore, alignment of payers and providers through alternative payment models may be beneficial for increasing quality and reducing costs.^53^ Moreover, global budgets were relatively successful in reducing healthcare utilisation in the Netherlands.^54^ Existing case studies underline the importance of accurate and timely data to support change processes,^7,44^ which seemed insufficiently acknowledged in the strategies of Bernhoven and the Beatrix hospital.

 The context of both hospitals was described extensively in this study. Previous studies on hospitals in the Netherlands have illustrated the importance of a local approach rather than using a blueprint strategy.^45,46^ Bernhoven and Beatrix showed clear contextual similarities and differences, but both were able to adjust a similar improvement rationale to local needs.

 Traditional concepts of quality improvement are considered linear interventions with a causal effect on outcomes, indicating that repeated interventions would lead to similar results. The results in this study demonstrate the importance of contextual factors. Improvement is not a single intervention in time but “an interdependent set of actions.”^12^ This study adds real-world evidence to the limited body of knowledge on hospital improvement strategies.

###  Implications

 Hospital-wide approaches for quality improvement and limiting costs may be successful. This study demonstrated that a convergent parallel mixed methods approach is useful for evaluating complex hospital interventions. Although context remains important for success, several general mechanisms were identified. We discern several implications for both policy-makers (macro-level) and hospital managers (meso-level).

 For hospital managers, a broad scope in engaging professionals, such as doctors and nurses, may improve effectiveness of the strategy on the operational level of the organisation. Hospital leadership should facilitate iterative cycles of improvement based on mutual goals. A drastic reorganisation of the hospital structure may be required to convert volume reductions into cost savings. The theoretical mechanism of shared savings appears, in practice, to require a delicate balance between a relative decrease in revenue and scaling down internal hospital costs.

 External policy reform may accelerate the shift towards appropriate care through alternative payment models, moving away from production-based incentives. Moreover, primary care providers may require compensation for additional burdens of substitution. Alternative or population-based payment models could provide fair allocation of resources, if risks for undertreatment and inefficiency can be restricted. This may aid the process of moving away from traditional siloes in healthcare, towards integrated care delivery.

 Recently, Bernhoven faced financial distress. The hospital was at the centre of the first wave of COVID-19 infections. In comparison to larger hospitals, smaller hospitals got fewer resources to compensate for additional costs. However, our analysis shows that the internal cost-structure of this hospital was already behind on its financial goals before the pandemic. The challenge of reorganising and scaling down hospital facilities should have a prominent emphasis in future strategies that aim to contribute to appropriate care. Our analyses showed that internal costs reductions lag volume reductions, resulting in relatively costly treatments. This may incentivise insurers to cut treatment prices and capitalize on lower levels of production.^55^ This ratchet effect is a concern for the longer-term sustainability of these strategies.

 We recommend an integrated approach to future research on hospital improvements. Baseline assessments of quality indicators, financial situation, and stakeholders’ perceptions before, during and after the runtime of the strategy implementation programme strengthen analyses. Longer-term effects of such programmes are currently unknown and research could offer important insights into longer-term sustainability. Further research may build on our findings to analyse how to consolidate and adapt improvement processes and mechanisms in the longer term.

## Conclusion

 We conducted an integrated analysis of mixed data sources to evaluate complex hospital improvement programmes. Two hospitals managed to (temporally) break the trend of annually increasing volumes, while maintaining quality of service delivery. However, long-term financial outcomes of these strategies raise concerns, since short-term cost reductions for both hospitals (substantially) lag behind volume reductions. Additional reorganisation efforts within and outside the hospital to scale down internal costs seem necessary to perpetuate the pursuit of improvement. These strategies underline the necessity of an approach customised to local context and which is subject to iterative improvement.

## Ethical issues

 Ethical approval was obtained at the Medical Research Ethics Committee Arnhem-Nijmegen, the Netherlands (Ref. 2019-5353).

## Competing interests

 JK was part of the transformation of both hospitals as external strategic advisor; no other relationships or activities that could appear to have influenced the submitted work.

## Funding

 This work was supported by the Ministry of Health, Welfare and Sport, The Hague, The Netherlands.

## Supplementary files


Supplementary file 1. Topic Guide.

Supplementary file 2. Overview of Respondents Semi-structured Interviews.

Supplementary file 3. Patient Satisfaction Scores in Bernhoven and Beatrix Hospital.

Supplementary file 4. Profit Margins Relative to Control Group Hospitals.
