# Supplementary material for: Improving Performance in Complex Surroundings: A Mixed Methods Evaluation of Two Hospital Strategies in the Netherlands
Source: Int J Health Policy Manag. 2023 May 6;12:7243. doi: 10.34172/ijhpm.2023.7243 (PMC10425645; doi:10.34172/ijhpm.2023.7243)
Supplement: Supplementary file 1 — Topic Guide. [file ijhpm-12-7243-s001.pdf]

Article title: Improving Performance in Complex Surroundings: A Mixed Methods Evaluation of Two Hospital Strategies in The Netherlands

Journal name: International Journal of Health Policy and Management (IJHPM)

Authors' information: Erik Wackers*, Simone van Dulmen, Bart Berden, Jan Kremer, Niek Stadhouders, Patrick Jeurissen

Radboud University Medical Center, Radboud Institute for Health Sciences, IQ healthcare, Nijmegen, The Netherlands.

(*Corresponding author: Erik.Wackers@radboudumc.nl)

Supplementary file 1. Topic Guide

The default topic guide was adapted to specific stakeholder groups.

Table S1. Topic guide semi structured interviews

**Start**: introductions, aim of the interview, informed consent
**Personal information**<br>What is your function in the organisation?<br>How long have you worked at this organisation?<br>How old are you?
**Context, aim and inception of the programme**<br>What does the programme mean to you?<br>Which elements are most important of the programme, according to you?<br>What was your opinion of the programme when it was introduced? And why?<br>What do you think of the programme now? Why?<br>Do you support the goals of the programme?<br>What is the impact of the programme on the quality of patient care, according to you?<br>What is the impact of the programme on the costs of care, according to you?

Implementation

How did the programme affect your day-to-day working activities?

How did you experience communications on progress of the programme during the process?

How were you involved in the improvement process?

What and who stimulated you to be involved?

Did you face barriers or limiting factors in your engagement? Could you elaborate?

Why was this organisation fit for such a programme?

What were the most important barriers that had to be overcome?

Are you proud of the organisation and how the programme progressed? Could you illustrate this?

How would you rate the programme on a scale from 1 to 10? And why?

What are, according to you, the most important prerequisites for hospitals to implement such an improvement programme?
