# Supplementary material for: Improving Performance in Complex Surroundings: A Mixed Methods Evaluation of Two Hospital Strategies in the Netherlands
Source: Int J Health Policy Manag. 2023 May 6;12:7243. doi: 10.34172/ijhpm.2023.7243 (PMC10425645; doi:10.34172/ijhpm.2023.7243)
Supplement: Supplementary file 2 — Overview of Respondents Semi-structured Interviews. [file ijhpm-12-7243-s002.pdf]

Article title: Improving Performance in Complex Surroundings: A Mixed Methods Evaluation of Two Hospital Strategies in The Netherlands

Journal name: International Journal of Health Policy and Management (IJHPM)

Authors' information: Erik Wackers*, Simone van Dulmen, Bart Berden, Jan Kremer, Niek Stadhouders, Patrick Jeurissen

Radboud University Medical Center, Radboud Institute for Health Sciences, IQ healthcare, Nijmegen, The Netherlands.

(*Corresponding author: Erik.Wackers@radboudumc.nl)

Supplementary file 1. Overview of Respondents Semi-structured Interviews

Table S2. Overview of respondents (Interviews were conducted between April 2019 and March 2020)

**Function**	**Bernhoven**	**Beatrix hospital**	**Respondents involved in both hospitals**	**Total *n***
Medical doctor	13	6	-	**19**
Nurse	3	2	-	**5**
General practitioner	4	3	-	**7**
Hospital manager	7	8	-	**15**
Primary care manager	1	2	-	**3**
Healthcare insurer	2	-	3	**5**
External consultant	-	-	4	**4**
Patient	5	4		**9**
**Total**	**35**	**25**	**7**	**67***

*62 respondents were interviewed, of which 5 held dual functions (e.g. medical doctors and managers)
