# Supplementary material for: Improving Performance in Complex Surroundings: A Mixed Methods Evaluation of Two Hospital Strategies in the Netherlands
Source: Int J Health Policy Manag. 2023 May 6;12:7243. doi: 10.34172/ijhpm.2023.7243 (PMC10425645; doi:10.34172/ijhpm.2023.7243)
Supplement: Supplementary file 3 — Patient Satisfaction Scores in Bernhoven and Beatrix Hospital. [file ijhpm-12-7243-s003.pdf]

Article title: Improving Performance in Complex Surroundings: A Mixed Methods Evaluation of Two Hospital Strategies in The Netherlands

Journal name: International Journal of Health Policy and Management (IJHPM)

Authors' information: Erik Wackers*, Simone van Dulmen, Bart Berden, Jan Kremer, Niek Stadhouders, Patrick Jeurissen

Radboud University Medical Center, Radboud Institute for Health Sciences, IQ healthcare, Nijmegen, The Netherlands.

(*Corresponding author: Erik.Wackers@radboudumc.nl)

Supplementary file 3. Patient Satisfaction Scores in Bernhoven and Beatrix Hospital

Figure S1. Patient satisfaction scores Beatrix 2008-2019

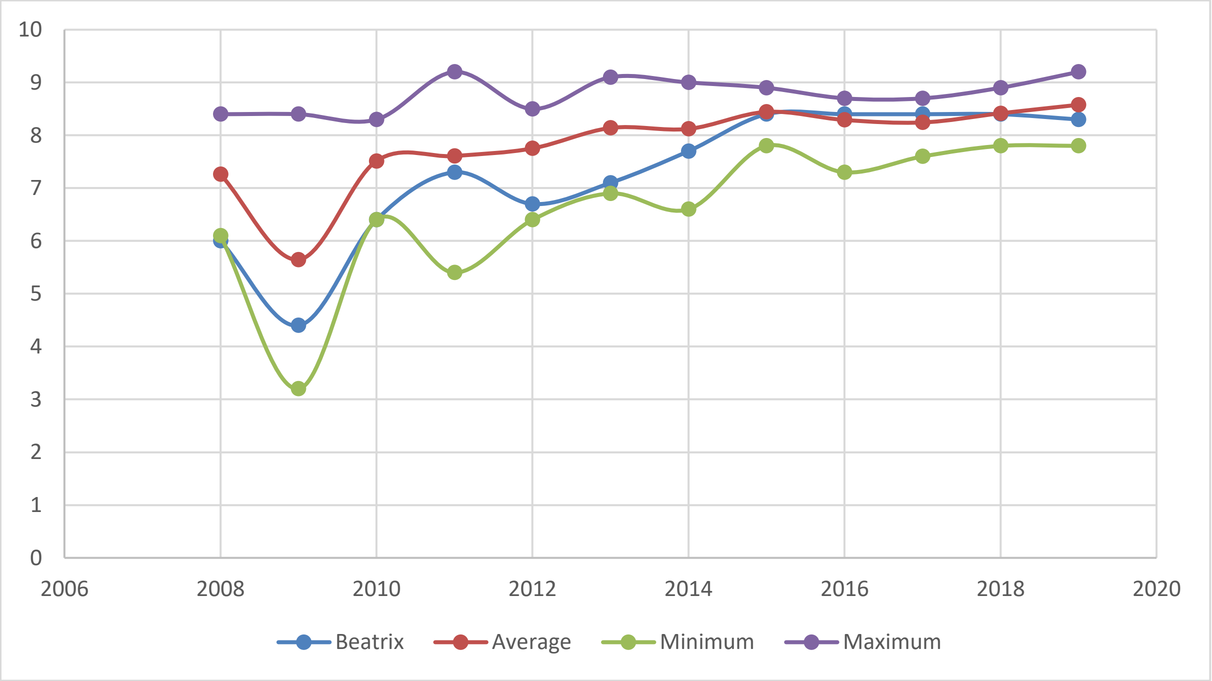

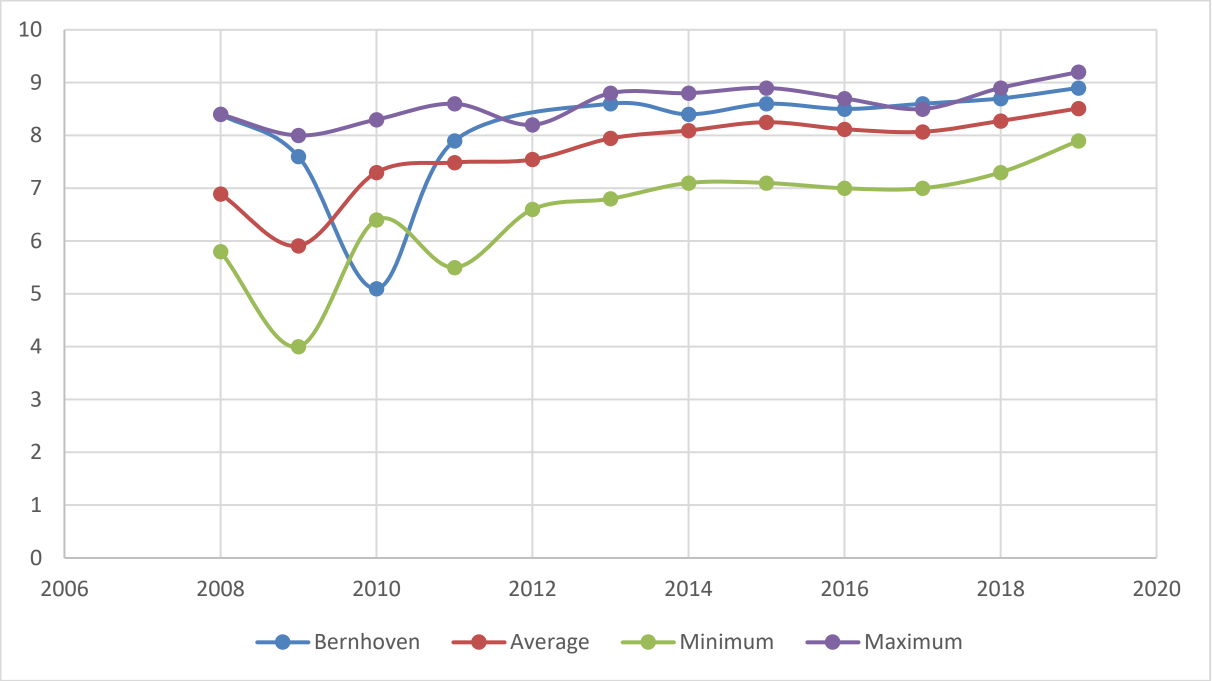

Figure S2. Patient satisfaction scores Bernhoven 2008-2019
