# Supplementary material for: Improving Performance in Complex Surroundings: A Mixed Methods Evaluation of Two Hospital Strategies in the Netherlands
Source: Int J Health Policy Manag. 2023 May 6;12:7243. doi: 10.34172/ijhpm.2023.7243 (PMC10425645; doi:10.34172/ijhpm.2023.7243)
Supplement: Supplementary file 4 — Profit Margins Relative to Control Group Hospitals. [file ijhpm-12-7243-s004.pdf]

Article title: Improving Performance in Complex Surroundings: A Mixed Methods Evaluation of Two Hospital Strategies in The Netherlands

Journal name: International Journal of Health Policy and Management (IJHPM)

Authors' information: Erik Wackers*, Simone van Dulmen, Bart Berden, Jan Kremer, Niek Stadhouders, Patrick Jeurissen

Radboud University Medical Center, Radboud Institute for Health Sciences, IQ healthcare, Nijmegen, The Netherlands.

(*Corresponding author: Erik.Wackers@radboudumc.nl)

Supplementary file 4. Patient Satisfaction Scores in Bernhoven and Beatrix Hospital

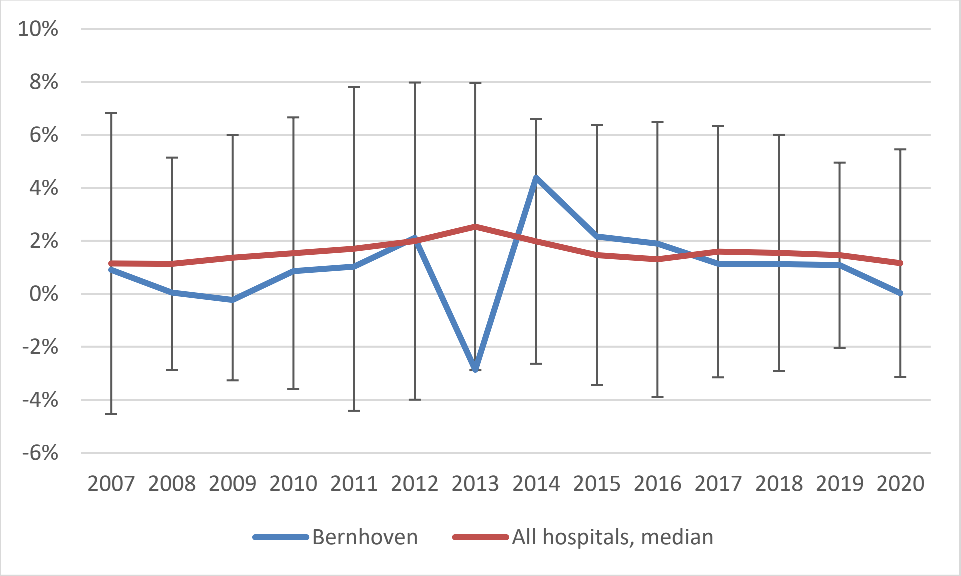

Figure S3: Profit margin Bernhoven relative to all hospitals. Error bars show minimum and maximum values.

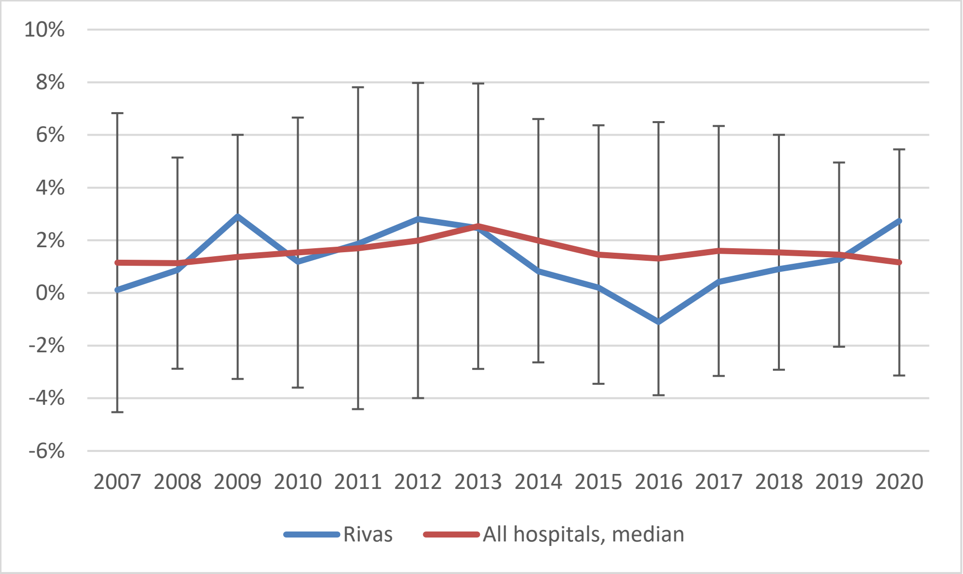

Figure S4. Profit margin Rivas care group (Beatrix) relative to all hospitals. Error bars show minimum and maximum values.
